# The emerging Nexus of STING signaling and ferroptosis: from mechanisms to therapeutic opportunities

**DOI:** 10.1038/s41420-026-03214-8

**Published:** 2026-07-07

**Authors:** Xixi Lin, Yihong Wen, Jiaheng Lang, Ziyu Zhu, Zijie Guo, Linbo Wang, Feiyang Ji, Lili Zhi, Jichun Zhou

**Affiliations:** 1 Department of Surgical Oncology, Affiliated Sir Run Run Shaw Hospital, Zhejiang University School of Medicine, Hangzhou, China; 2 Zhejiang Key Laboratory of Biotherapy, Hangzhou, China

**Keywords:** Cell death, Cell signalling

## Abstract

The cyclic GMP-AMP synthase-stimulator of interferon genes (cGAS-STING) pathway, a cornerstone of innate immunity, and ferroptosis, an iron-dependent form of regulated cell death driven by lipid peroxidation, have traditionally been studied as distinct entities. However, emerging evidence reveals a complex and bidirectional crosstalk between these two pathways with profound implications for disease pathogenesis and therapy. This review systematically synthesizes the current understanding of the multifaceted interactions between the cGAS-STING pathway and ferroptosis. We detail the mechanisms by which STING signaling promotes ferroptosis through iron metabolism (e.g., NCOA4-mediated ferritinophagy), lipid peroxidation (e.g., via ACSL4 interaction), and GPX4 autophagic degradation. Conversely, we explore how ferroptosis, through mitochondrial DNA release and lipid peroxidation products, can activate the cGAS-STING pathway, amplifying immune and inflammatory responses. A novel, non-canonical role for mitochondrially-localized cGAS in suppressing ferroptosis independent of STING is also highlighted, adding a layer of complexity to this interplay. We consolidate evidence of this crosstalk across a spectrum of diseases, including cancer, infectious diseases, neurodegenerative disorders, and ischemia-reperfusion injuries. In cancer, leveraging this interplay—particularly by inducing ferroptosis to activate STING-dependent anti-tumor immunity—presents promising therapeutic strategies. In contrast, for inflammatory and organ injuries, concurrent inhibition of both pathways may mitigate damage. This review underscores the STING-ferroptosis axis as a critical regulatory node and a promising frontier for developing novel therapeutic interventions across diverse human diseases.

## Introduction

In 2012, the laboratory of Brent R. Stockwell formally defined a novel form of iron-dependent programmed cell death as ferroptosis [1]. Since its identification, ferroptosis has been extensively investigated over the past decade across diverse disease contexts, including neurodegenerative disorders, ischemia-reperfusion injury, organ failure, and tumor therapy resistance. The ferroptosis pathway presents numerous therapeutic targets; consequently, significant research efforts and clinical trials have focused on targeting key molecules within this pathway, offering novel strategies and directions for treating currently incurable conditions [2]. Furthermore, increasing attention has been directed towards the role of ferroptosis in infectious and immune-related diseases [3]. Notably, the cyclic GMP-AMP synthase (cGAS)-STING pathway is recognized as a critical bridge linking damaged DNA to immune activation. It plays a pivotal role in innate immunity against exogenous infections, such as those caused by bacteria and viruses, and has recently emerged as a major research focus. Its contributions to human inflammatory diseases and cancer have been extensively studied [4, 5]. The cGAS-STING pathway is responsible for sensing aberrant DNA and primarily promotes immune responses through two major mechanisms: eliciting type I interferon (IFN) release via IRF3 and facilitating the production of pro-inflammatory cytokines through NF-κB activation [6].

Substantial evidence now demonstrates a crosstalk between ferroptosis and the cGAS-STING pathway. Beyond revealing phenotypic findings, numerous studies have delved into the specific molecular mechanisms underlying this interaction. This review systematically summarizes the currently established interplay between ferroptosis and the cGAS-STING pathway, including its detailed mechanisms, and synthesizes the implications of this crosstalk for the pathogenesis and potential treatment of various diseases. We begin by providing a concise mechanistic overview of the STING pathway and ferroptosis.

### Overview of ferroptosis

**Fig. 1:**
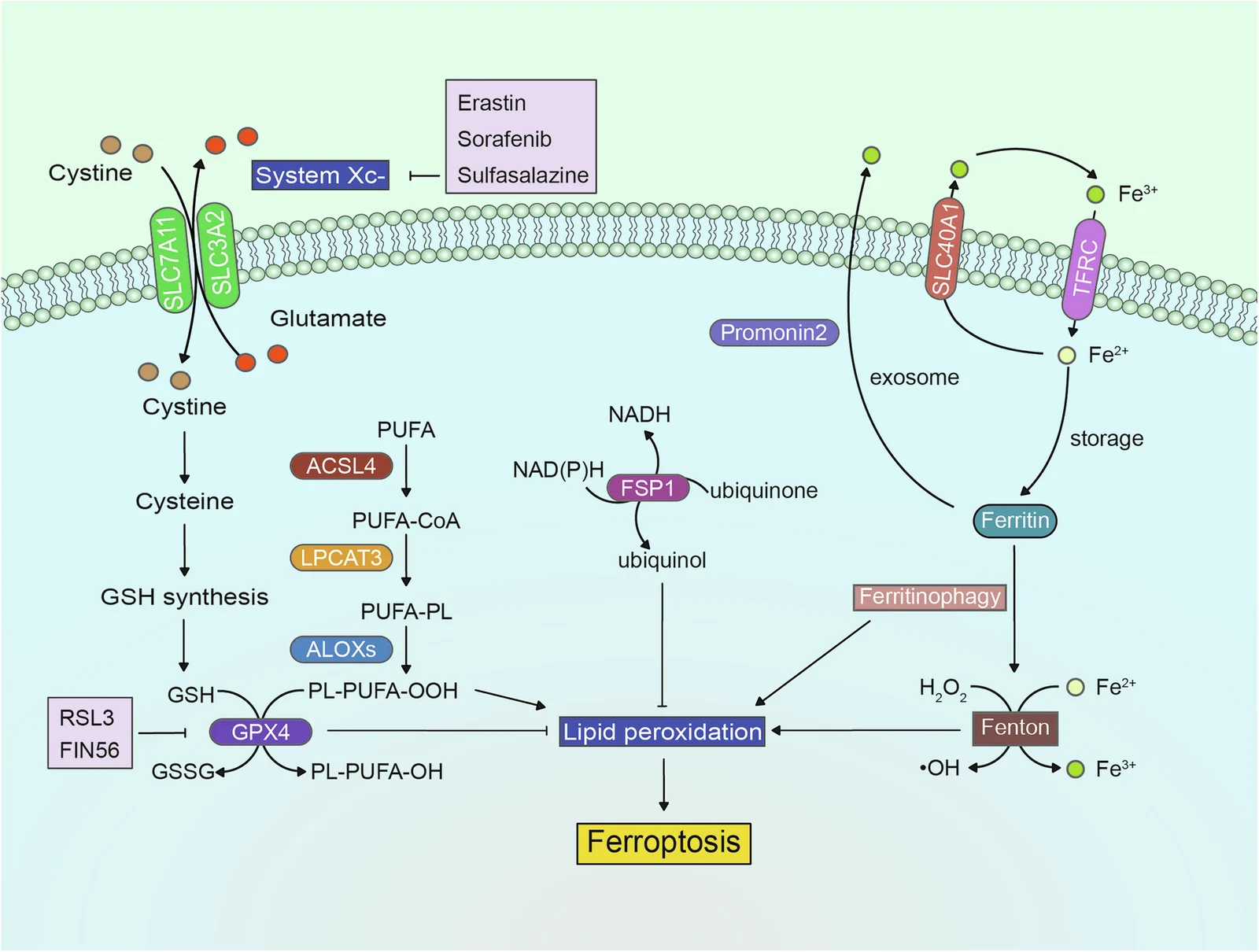
Overview of the ferroptosis pathway. The executioner of ferroptosis is iron-dependent excessive lipid peroxidation. During lipid peroxidation, PUFA is oxidized to PL-PUFA-PLOOH, under the catalysis of ACSL4, LPCAT3 and ALOXs, thus fueling ferroptosis. Ferroptosis is mainly controlled by two metabolic pathways, namely the system Xc-–GSH–GPX4 axis and NAD(P)H/ FSP1/ CoQ10 axis. The cystine/glutamate transporter (known as system Xc−, composed of SLC7A11 and SLC3A2 subunits) imports cystine while exporting glutamate and can be inhibited by erastin, sorafenib and SAS. Then imported cystine is reduced to cysteine, which is crucial for biosynthesis of GSH, and ultimately leads to the reduction of oxidized phospholipids (PL-PUFA-OOH) to the corresponding alcohols (PL-PUFA-OH) by GPX4, which can be inhibited by RSL3 and FIN56. NAD(P)H/ FSP1/ CoQ10 axis converts NAD(P)H to NAD(P)+ while reducing ubiquinone (CoQ10) to ubiquinol, which prevents lipid peroxidation. Iron plays an indispensable role in regulating ferroptosis.Fe3+ is transported into cells by TFRC and turns to Fe2 + , stored by ferritin, which is degraded via ferritinophagy and encapsulated and transported out of the cells mediated by Prominin2 in the form of an exosome. Iron can also be exported by SLC40A1. Furthermore, the Fenton reaction generates hydroxyl radical (•OH) via the reaction of ferrous ions (Fe2 + ) and H2O2, thus contributing to lipid peroxidation.

Ferroptosis is a form of regulated cell death (RCD) distinct from apoptosis, necrosis, and pyroptosis. Its core definition is cell death driven by uncontrolled lipid peroxidation [7], a process inhibitable by both iron deprivation and lipophilic radical-trapping antioxidants (RTAs) [8]. Mechanistically, ferroptosis is regulated by the canonical glutathione peroxidase 4 (GPX4) pathway and by metabolism-independent mechanisms, ultimately being driven by excessive lipid peroxidation. We next delineate the principal regulatory mechanisms of ferroptosis (Fig. 1).

The System Xc⁻–GSH–GPX4 Axis constitutes the core metabolic regulatory pathway for ferroptosis. System Xc⁻ is a sodium-independent cystine/glutamate antiporter, a transmembrane protein complex composed of the SLC7A11 (xCT) and SLC3A2 subunits [9, 10]. Its primary function is to import cystine into the cell while exporting glutamate to the extracellular space. Its activity can be inhibited by compounds such as erastin, sorafenib, and sulfasalazine. Intracellular cystine, the oxidized form of cysteine, is reduced to cysteine. Cysteine is essential for glutathione (GSH) synthesis, serving not only as a precursor but also as the rate-limiting substrate [11, 12]. GSH, a tripeptide composed of glutamate, cysteine, and glycine, is a crucial intracellular reductant. It acts as an electron donor for glutathione peroxidases (GPXs), enabling them to reduce peroxides, including hydrogen peroxide [8]. GPX4, a selenoprotein member of the GPX family inhibitable by RSL3 and FIN56, reduces phospholipid hydroperoxides (PLOOHs) while oxidizing GSH to glutathione disulfide (GSSG), thereby suppressing ferroptosis [11].

Beyond the classical System Xc⁻–GSH–GPX4 pathway, GPX4-independent regulatory axes play significant roles in ferroptosis. The NAD(P)H/FSP1/CoQ₁₀ Axis is Identified as a major GPX4-independent axis [2, 8, 13]. Ferroptosis suppressor protein 1 (FSP1), primarily localized to lipid droplets and the plasma membrane [14], functions as an oxidoreductase responsible for detoxifying deleterious lipid hydroperoxides [15]. Utilizing reducing equivalents from NAD(P)H (converted to NAD(P)⁺), FSP1 reduces ubiquinone (Coenzyme Q₁₀, CoQ₁₀) to ubiquinol [16], acting as a lipophilic RTA, directly quenching lipid peroxyl radicals (PLOO•) or indirectly preventing lipid peroxidation via regeneration of vitamin E (α-tocopherol) [2, 13].The GTP cyclohydrolase 1 (GCH1)/tetrahydrobiopterin (BH4) pathway was established in 2020 as a GPX4-independent ferroptosis suppressor pathway [17]. GCH1, the rate-limiting enzyme in BH4 biosynthesis, inhibits ferroptosis through its metabolite BH4, a potent radical scavenger. Dihydroorotate dehydrogenase (DHODH) was first identified in 2021 to exert ferroptosis inhibition independently of the canonical GPX4 pathway [18, 19]. DHODH mitigates ferroptosis within mitochondria by modulating dihydroorotate oxidation and ubiquinol regeneration at the inner mitochondrial membrane.

Iron metabolism imbalance is a central driver of ferroptosis. Cells maintain iron homeostasis through tightly regulated uptake, storage, and efflux. Transferrin receptor 1 (TFR1) mediates iron influx, while ferroportin 1 (FPN1) mediates iron efflux. Ferritin stores iron in an inert form; its autophagic degradation (ferritinophagy), mediated by the receptor NCOA4, releases Fe²⁺ to fuel the Fenton reaction [20]. Increased intracellular labile iron levels, resulting from perturbations in iron transport or ferritinophagy leading to expansion of the labile iron pool (LIP), can drive ferroptosis [8].

Excessive lipid peroxidation is the hallmark of ferroptosis. During lipid peroxidation, phospholipid hydroperoxides (PLOOHs), lipid-based reactive oxygen species (ROS) generated from oxidized polyunsaturated fatty acids (PUFAs), act as the terminal executioners [8]. PUFAs are esterified to PUFA-CoA by ACSL4 and subsequently incorporated into phospholipids (primarily phosphatidylethanolamine, PE) by LPCAT3, forming PUFA-phospholipids (PUFA-PLs). Under the action of lipoxygenases (ALOXs, particularly the ALOX15/ACSL4 complex) or radicals (e.g., •OH), PUFA-PLs are oxidized to phospholipid hydroperoxides (PL-PUFA-OOH), directly compromising membrane integrity [7, 21].

### Overview of the cGAS-STING pathway

**Fig. 2:**
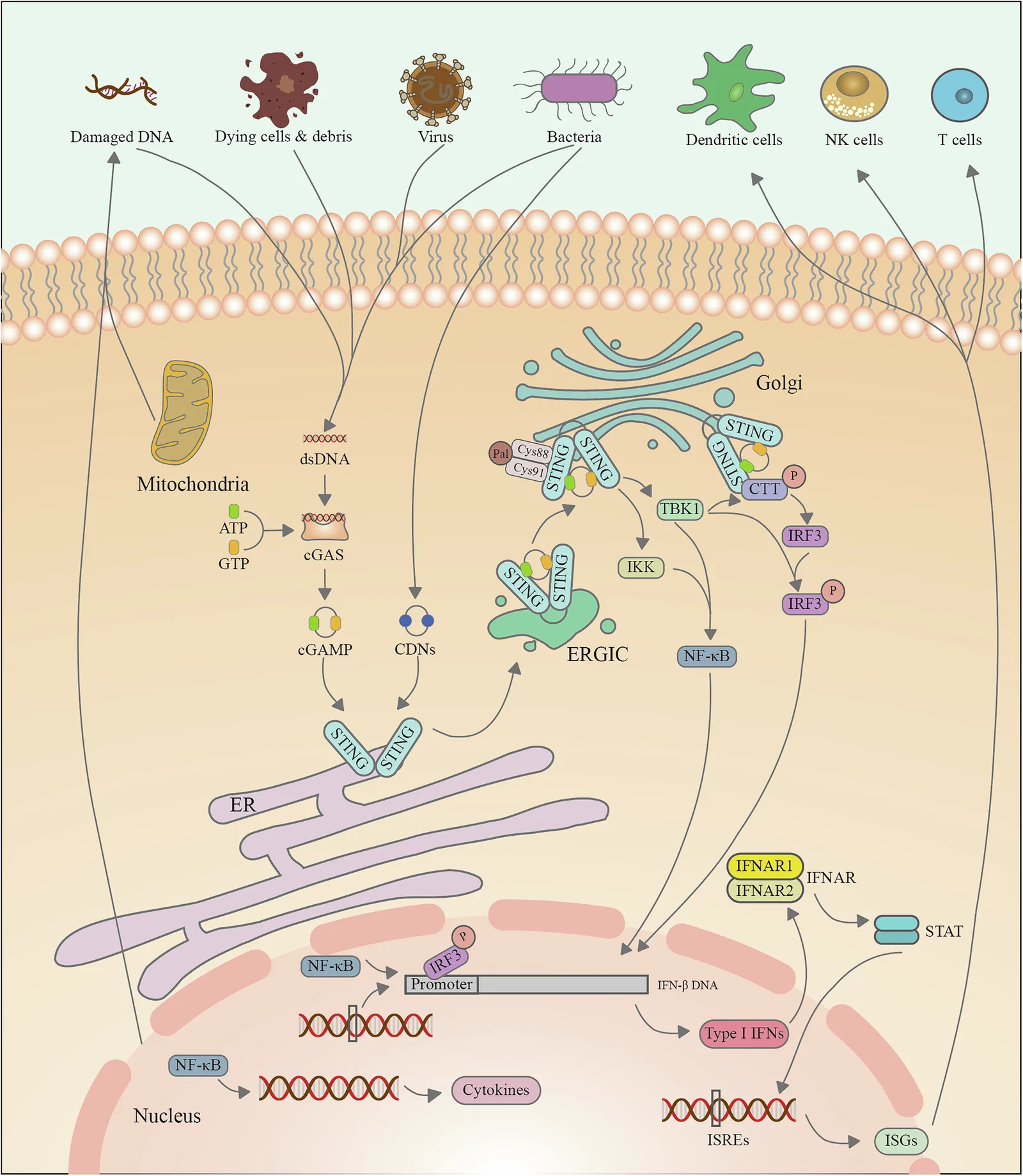
Overview of the CGAS-STING pathway. In response to abnormal cytoplasmic DNA, cGAS triggers an immune response by producing cGAMP, which binds and activates STING. In addition, CDNs can directly activate STING. STING oligomerizes in the ER and migrates to the Golgi apparatus through the ERGIC. In the Golgi apparatus, STING is palmitoylated at Cys88 and Cys91, leading to the recruitment of TBK1 and IRF3. At the same time, STING also recruits IKKε to activate NF-κB. Ultimately, NF-κB and IRF3 enhance the expression and secretion of type I IFN, which activates IFNAR1/2, leading to the expression of ISGs via STAT, thus activating DC, T lymphocytes, and NK cells to target and kill cells. Moreover, NF-κB also contributes to the expression of pro-inflammatory cytokines.

As an evolutionarily highly conserved endoplasmic reticulum (ER) transmembrane protein, STING (stimulator of interferon genes) has garnered significant attention in recent years for its pivotal role in innate immunity and immune responses against pathogens [22, 23]. The cGAS-STING pathway primarily functions to detect aberrant cytosolic double-stranded DNA (dsDNA) and promote immune responses, chiefly executed through two canonical downstream signaling axes: the IRF3 and NF-κB pathways [23]. We provide a concise overview of the cGAS-STING pathway activation and signal transduction (Fig. 2).

DNA substrates, whether self-derived or non-self (e.g., originating from invading pathogens or damaged host cells), can activate the DNA sensor cGAS [24]. Activated cGAS synthesizes the second messenger 2’,3’-cyclic GMP-AMP (2'3’-cGAMP) from ATP and GTP, which subsequently binds to and activates STING [25]. In addition to 2'3’-cGAMP, bacterial cyclic dinucleotides (CDNs) can also directly activate STING [26]. Upon activation within the ER, STING undergoes oligomerization and traffics through the ER-Golgi intermediate compartment (ERGIC) to the Golgi apparatus [27].

At the Golgi, STING is palmitoylated at cysteine residues Cys88 and Cys91 [28]. This palmitoylation facilitates the recruitment of TANK-binding kinase 1 (TBK1). TBK1 then phosphorylates STING, enabling STING to recruit interferon regulatory factor 3 (IRF3). TBK1 directly phosphorylates IRF3, leading to its dimerization and translocation to the nucleus, where it binds to the interferon-beta (IFN-β) promoter region. Concurrently, activated STING also binds to and stimulates IκB kinase epsilon (IKKε), resulting in the activation of the transcription factor nuclear factor kappa B (NF-κB) [22]. NF-κB and IRF3 cooperatively form an enhanceosome complex at the IFN-β promoter, maximizing the expression and secretion of type I interferons (IFNs). These secreted IFNs bind to the interferon alpha/beta receptor 1 and 2 (IFNAR1/2) [23], inducing autophosphorylation of tyrosine kinase 2 (TYK2) and Janus-activated kinase 1 (JAK1). This activates signal transducer and activator of transcription (STAT) proteins 1 and 2, which dimerize, translocate to the nucleus, bind to interferon-stimulated response elements (ISREs), and induce the expression of interferon-stimulated genes (ISGs) [29]. This cascade ultimately promotes the activation and migration of immune cells, including dendritic cells (DCs), T lymphocytes, and natural killer (NK) cells, to exert their effector functions [30, 31]. Beyond its cooperative role with IRF3 in type I IFN production, NF-κB also transcriptionally upregulates key pro-inflammatory cytokines such as TNF and IL-6 [23], playing crucial roles in host defense responses.

## The interplay between the cGAS-STING pathway and ferroptosis

### The cGAS-STING promotes ferroptosis

Increasing evidence has confirmed a mutually promotive interplay between the cGAS-STING pathway and ferroptosis in various diseases, which are compiled in Table 1. Neuropeptide substance P attenuates colitis’ inflammatory levels by inhibiting cGAS/STING-mediated ferroptosis [32]. Ferroptosis triggered by the cGAS-STING signaling pathway is involved in the process of polystyrene microplastics-induced pulmonary fibrosis [33]. The investigation revealed that Snord3a expression was elevated in renal tubules under conditions of acute kidney injury (AKI). The regulatory function of Snord3a on the STING signaling axis, by fostering the transcription of the STING gene, contributes to ferroptosis, underscoring the significance of Snord3a as a promising prognostic and therapeutic target for AKI [34]. In the context of bleomycin-induced lung injury, the transportation of Fcn B by exosome from alveolar macrophages is observed to exacerbate ferroptosis in lung epithelial cells via the cGAS-STING signaling pathway [35]. A study on sepsis-induced cardiomyopathy (SIC) revealed that increased expression of islet cell autoantigen 69 (ICA69) triggers STING generation, leading to intracellular lipid peroxidation and ultimately culminating in ferroptosis and cardiac injury. ICA69 deficiency alleviated inflammation and ferroptosis in Ferrintinophagymurine investigation hearts and macrophages of LPS-induced mice, suggesting that targeting ICA69 represents a potentially promising therapeutic strategy for SIC [36]. In tendon stem cells (TSCs), hydrogen peroxide (H₂O₂) activates the cGAS-STING pathway, facilitating the onset of ferroptosis. This process is potentially mediated by mitophagy. In the murine model of tendinopathy, disruption of cGAS mitigates tendon injury by suppressing ferroptosis [37]. Another study also revealed that suppression of ferroptosis via inhibition of the cGAS-STING pathway plays a vital role in berberine alleviating tendinopathy [38]. STING knockdown alleviates lipid toxicity-induced myocardial ferroptosis and reduces cardiac injury both in vivo and in vitro, identifying STING as a novel therapeutic target for lipotoxic cardiomyopathy [39]. A lupus nephritis study demonstrates that STING activation promotes ferroptosis and inflammatory responses via the TBK1/NF-κB pathway and STING inhibition mitigates ferroptosis, renal damage, and inflammatory cell infiltration in mice [40]. In the investigation of cervical cancer, PIN1 suppresses the cGAS-STING pathway to inhibit ferroptosis, thereby enhancing tumor cell proliferation and invasion [41]. STING silencing in the chronic phase of traumatic brain injury (TBI) confers anti-ferroptotic effects comparable to moderate-intensity treadmill running [41]. In acute myeloid leukemia (AML) therapy, PSTKi potentiates cGAS-STING activation to exacerbate ferroptosis in leukemia stem cells, eliminating residual cancer cells after chemotherapy and preventing relapse [42]. Manganese chloride amplifies cGAS-STING-mediated IFN-I signaling to disrupt dihydroorotate dehydrogenase (DHODH)-dependent ferroptosis defense, proposing a potential adjuvant strategy for antitumor regimens [43]. IFN-β produced by the cGAS-STING activation elevates intracellular Fe²⁺ and lipid peroxidation, meanwhile depleting GSH, inducing ferroptosis-associated transcriptomic dysregulation [44]. Consequently, IFN-β synergizes with RSL3 to enhance ferroptosis, potently suppressing tumor growth in HT1080 spheroids and xenograft models.

**Table 1:** The crosstalk between STING and ferroptosis in diseases.

Diseases	STING promotes ferroptosis	Ferroptosis promotes STING
Colitis	32	–
Pulmonary fibrosis	33	–
Bleomycin-induced lung injury	35	–
Acute kidney injury (AKI)	34	–
Lupus nephritis	40	–
Cardiomyopathy	36,39,50,51	–
Tendinopathy	37,38	–
Acute myeloid leukemia (AML)	43	–
CANCER	41,44,45,49	52–58
Liver injury	46	59
Parkinson’s disease (PD)	49,50	–
Alzheimer’s disease (AD)	–	61
Traumatic brain injury (TBI)	42	–

Ginsenoside Rd protects against carbon tetrachloride (CCl₄)-induced acute liver injury (ALI) by suppressing ferroptosis through inhibition of the cGAS-STING pathway [45]. Convergent studies in parkinson’s disease (PD) models demonstrate that targeting the cGAS-STING-ferroptosis axis ameliorates motor deficits and promotes recovery of dopaminergic neurons in the substantia nigra [46, 47]. In vitro, evidence indicates that METTL14 overexpression reduces ferroptosis, mediated by downregulation of cGAS-STING signaling [46]. And dingzhen pills attenuate neuronal ferroptosis and neuroinflammation via suppression of the cGAS-STING pathway [47]. As for tumor therapy, mtDNA G-quadruplex-binding ligand, BYB, induces autophagic-dependent ferroptosis through cGAS-STING pathway activation, revealing its therapeutic potential [48]. Swimming exercise mitigates ferroptosis in doxorubicin (DOX)-induced cardiomyopathy (DIC) by elevating miR-17-3p to activate the KEAP1/NRF2 axis while suppressing the cGAS-STING signaling [49]. For tourniquet (TQ)-induced myocardial injury during total knee arthroplasty (TKA)—a consequence of limb ischemia-reperfusion, esketamine attenuates ferroptosis, which can be rescued by STING overexpression [50].

### Ferroptosis promotes the cGAS-STING pathway

In GPX4 depletion or high-iron diet-mediated ferroptosis, 8-OHG release activates the STING pathway, driving macrophage infiltration, activation, and migration to accelerate pancreatic ductal adenocarcinoma (PDAC) progression [51]. Mechanistically, ferroptosis-induced mtDNA leakage into the cytosol activates the cGAS-STING pathway in tumor cells—a targetable vulnerability exploited by Exo@MnIO&BG, a multifunctional exosome that disrupts intracellular iron homeostasis and redox balance to amplify ferroptosis and enhance cGAS-STING-based immunotherapy [52], which mtDNA-centric mechanism further inspired HBMn-FA, a mitochondrial DNA-guided nanoplatform for tumor immunotherapy [53]. Similarly, a novel radiosensitizer augments radiotherapy efficacy by ferroptosis cascade-amplified activation of the cGAS-STING pathway via damaged DNA and Mn²⁺ [54]. A study reveals that ferroptosis in head and neck squamous cell carcinoma(HNSCC) suppresses tumor growth and activates the STING-IFN-I axis, recruiting and maturing dendritic cells [55]. Crucially, IFN-I reinforces ferroptosis by suppressing the xCT-GPX4 antioxidant system. Leveraging this feedback loop, combined ferroptosis inducers and STING agonists significantly inhibit HNSCC growth, amplify ferroptosis, and enhance dendritic cell infiltration. Suppression of ferroptosis inhibits STING signaling in liver macrophages, thereby promoting M2 polarization and ameliorating spontaneous liver injury, fibrosis, and tumorigenesis induced by TAK1 deficiency [56]. In tumor- associated macrophage reprogramming strategies, the dihydroartemisinin (DHA)-loaded MOF nanoreactor DHA@MIL-101 activates ferroptosis elevation and STING signaling, promoting TAMs remodeling towards M1 phenotype [57]. In alzheimer’s disease(AD), ferroptotic damage-triggered STING signaling plays a significant role in neuroprotection exerted by silymarin in sporadic AD models by attenuating ferroptosis and subsequent STING-mediated neuroinflammation [58]. As summarized in Table 1, the role of ferroptosis in activating the cGAS-STING pathway has been primarily established through studies in cancer.

### Mitochondrial cGAS: A non-canonical pathway for ferroptosis inhibition

Conventionally recognized as a cytosolic DNA sensor, cGAS activates the STING pathway via cGAMP synthesis to induce immune responses and promote ferroptosis. However, a recent study reveals that cGAS localizes to the mitochondrial outer membrane through a mitochondrial targeting sequence (MTS), establishing a STING-independent ferroptosis-suppressive pathway that fundamentally reshapes our understanding of cGAS functionality. The C-terminal domain of cGAS (aa 161-522) binds the GTPase domain of dynamin-related protein 1 (DRP1), promoting DRP1 oligomerization to inhibit mitochondrial ROS production and ferroptosis, thereby facilitating tumorigenesis [59]. This discovery suggests that disrupting cGAS-MTS localization (e.g., via MTS-competing peptides) or blocking cGAS-DRP1 interaction may specifically induce tumor ferroptosis, offering novel therapeutic strategies. Critically, mitochondrial cGAS represents a non-canonical, STING-independent regulatory axis for ferroptosis control.

### Cross-talk at the intersection of iron metabolism

#### Ferritinophagy

**Fig. 3:**
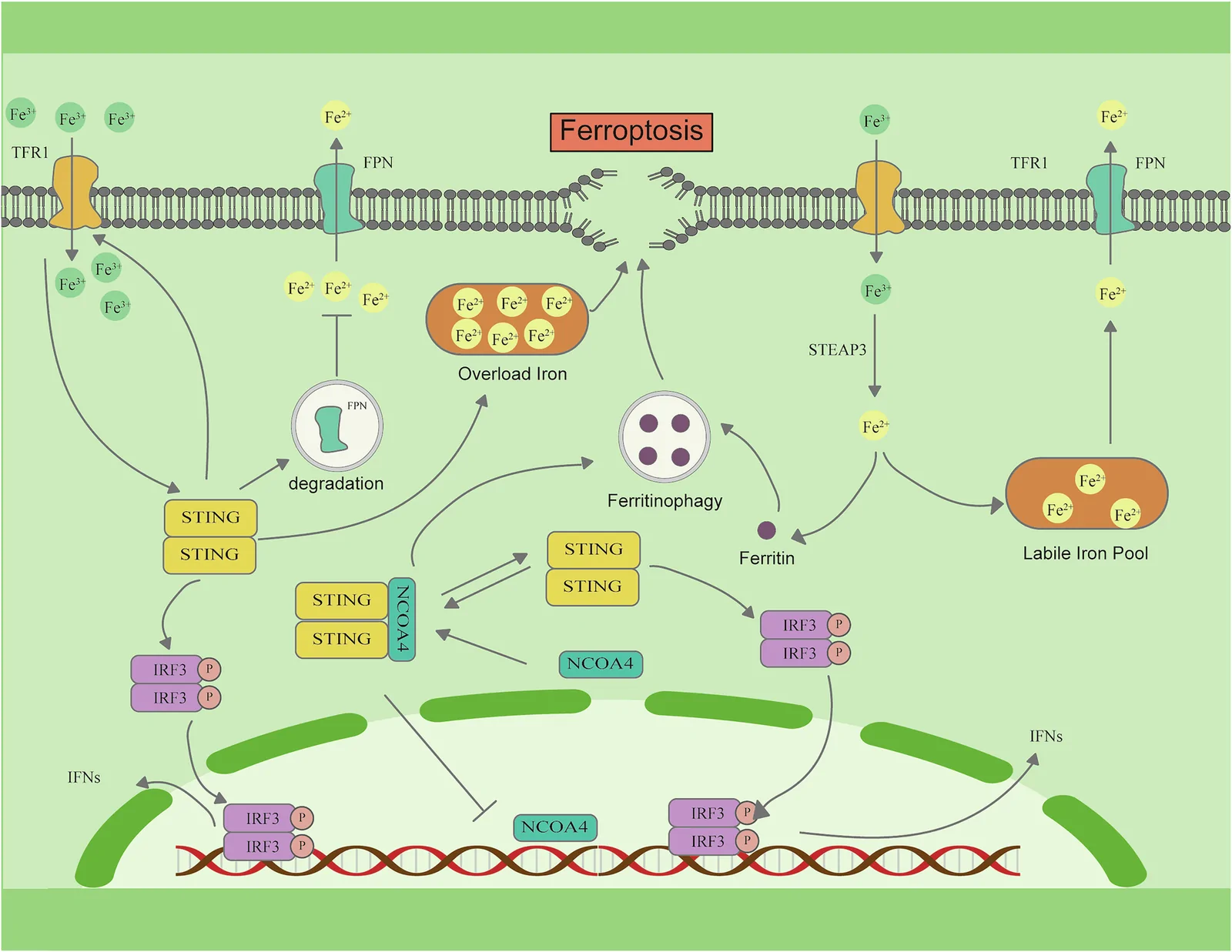
The Interplay via Iron Metabolism Pathways. Iron metabolism—comprising iron influx, efflux, and ferritinophagy—mediates the crosstalk between ferroptosis and the STING pathway. (1)Iron Influx: STING promotes the expression of TFR1 and facilitates TFR1-mediated iron influx. However, excessive intracellular iron overload resulting from upregulated TFR1 triggers cGAS/STING-dependent inflammatory responses. (2)Iron Efflux: STING enhances the ubiquitin-proteasomal degradation of FPN1, leading to reduced FPN1 protein levels and impaired iron efflux, thereby promoting ferroptosis. (3)NCOA4-Mediated Ferritinophagy: STING interacts with NCOA4, stabilizing STING dimerization. This interaction diminishes the nuclear localization of NCOA4, impairing its co-regulatory function as a transcriptional coactivator and subsequently triggering ferritinophagy-mediated ferroptosis.

Ferritinophagy, a selective autophagy process specifically degrading ferritin to release iron, plays a pivotal role in intracellular iron homeostasis. Ferritinophagy is mediated by the cargo receptor nuclear receptor coactivator 4 (NCOA4), which binds ferritin and facilitates its delivery to autophagosomes. Subsequent autophagosome-lysosome fusion degrades ferritin, releasing Fe²⁺ into the cytosol to complete ferritinophagy. As shown in Fig. 3, mounting evidence reveals reciprocal regulation between the STING pathway and ferritinophagy, with demonstrated NCOA4-STING interactions.

A study revealed that severe mtDNA damage activates the cGAS-STING axis, triggering ferritinophagy-driven ferroptosis– a mechanism exploited by the H₂S-responsive iridium(III) complex NA-Ir for cancer therapy [60]. In cerebral ischemia-reperfusion injury (CIRI), ferritinophagy activates cGAS-STING signaling, exacerbating neuroinflammation via ferroptosis-dependent microglial oxidative stress and inflammatory responses [61]. During the early stage of CIRI, NCOA4-mediated ferritinophagy critically contributes to brain damage. Inhibition of the cGAS-STING pathway attenuates NCOA4-driven ferritinophagy, thereby ameliorating cerebral injury [62]. STING promotes ferroptosis in acute kidney injury through NCOA4-mediated ferritinophagy [63, 64], and the traditional Chinese formula Yi-Qi-Jian-Pi-Xiao-Yu (YQJPXY) attenuates cisplatin-induced nephrotoxicity by inhibiting the STING-NCOA4-ferritinophagy-ferroptosis cascade [64]. African swine fever virus protein A151R induces STING carbonylation by enhancing NCOA4-dependent ferritinophagy, thereby suppressing TBK1-IRF3 signaling and IFN-β production [65]. Mechanistic studies reveal that in sepsis-activated macrophages, residues Q237, E316, and S322 within STING’s CBD domain interact with NCOA4’s coiled-coil domain. This interaction stabilizes STING dimers, enhances inflammatory responses, reduces NCOA4 nuclear localization impairing its co-regulatory function, and triggers ferritinophagy-mediated ferroptosis—exacerbating sepsis-induced intestinal injury, which cascade is inhibited by HET0016 [66]. In ox-LDL-injured HUVECs, STING upregulates NCOA4 expression, promotes its nuclear translocation, and binds NCOA4 to modulate ferritinophagy, thereby facilitating LOX-1-induced ferroptosis—revealing novel therapeutic avenues for atherosclerosis [67]. cGAS knockout in alveolar type 2 (AT2) cells disrupts NCOA4-ferritin binding, suppressing ferritinophagy and ameliorating PM2.5-induced lung injury [68].

#### Iron transport

Transferrin receptor 1 (TFR1) and ferroportin 1 (FPN1) maintain cellular iron homeostasis by mediating iron influx and efflux, respectively, serving as critical regulators of ferroptosis. Emerging evidence implicates that the STING pathway plays a role in ferroptosis mediated by TFR1/FPN1-dependent iron dysregulation. Excessive intracellular iron load driven by TFR1 overexpression not only induces oxidative stress and sensitizes chondrocytes to ferroptosis but also triggers cGAS/STING-mediated inflammation by promoting mitochondrial damage and mtDNA release. Conversely, TFR1 inhibition suppresses cGAS/STING-driven inflammation. This study further demonstrates that STING activation promotes TFR1 expression and iron influx, suggesting a self-perpetuating loop exacerbating osteoarthritis pathogenesis [69]. FPN1 facilitates cellular iron export; its functional impairment causes pathological iron accumulation. In diabetic kidney disease (DKD), STING-deficient mice (STING^gt/gt^) exhibit reduced ubiquitin-proteasomal degradation of FPN1, thereby increasing FPN1 protein levels, attenuating ferroptosis, and revealing a therapeutic strategy for DKD [70].

### Cross-talk at the intersection of lipid metabolism

Lipid metabolism plays a critical role in ferroptosis, with excessive lipid peroxidation serving as its hallmark. During lipid peroxidation, phospholipid hydroperoxides (PLOOHs), lipid-based reactive oxygen species (ROS) generated from the oxidation of polyunsaturated fatty acids (PUFAs) mediated by ACSL4, LPCAT3, and ALOXs, act as the executioners of ferroptosis [8]: PUFA oxidation involves the conjugation of PUFA with coenzyme A (CoA) catalyzed by ACSL4 to form PUFA-CoA. LPCAT3 then facilitates re-esterification to generate PUFA-phospholipids (PUFA-PLs), which are subsequently oxidized by ALOXs to form PL-PUFA-PLOOH [7, 21].

**Fig. 4:**
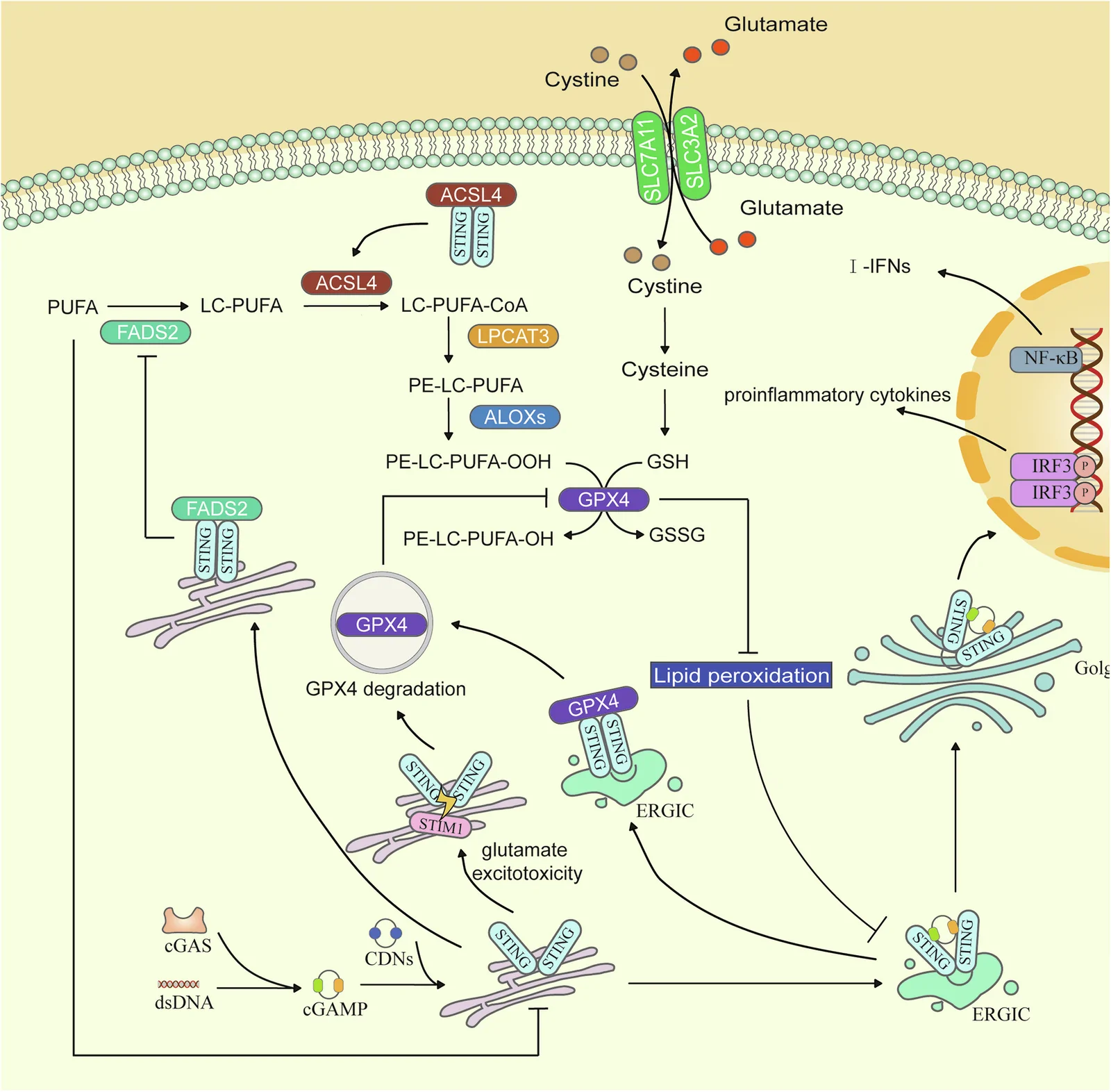
The interplay via lipid metabolism and GPX4 homeostasis. (1) STING directly binds to ACSL4 to promote lipid peroxidation. Furthermore, it interacts with FADS2, suppressing the production of derivatives such as long-chain polyunsaturated fatty acids (LC-PUFAs). Notably, PUFAs can, in turn, inhibit STING activity. (2) Inhibition of GPX4-mediated lipid peroxidation suppresses the translocation of STING from the endoplasmic reticulum to the Golgi apparatus, thereby blocking its downstream signaling. Additionally, STING can directly bind to GPX4. The resulting STING-GPX4 complex initially localizes to the ER-Golgi intermediate compartment (ERGIC) and is subsequently trafficked to autophagosomes, leading to GPX4 degradation. Separately, glutamate excitotoxicity triggers the dissociation of STING from STIM1, which induces autophagic degradation of GPX4 and promotes ferroptosis.

Multiple studies have elucidated the relationship between lipid peroxidation and the STING pathway, as shown in Fig. 4. In HSV-1-infected mice, lipid peroxidation induced by GPX4 inhibition impeded STING activation by suppressing its palmitoylation at Cys88/Cys91. This prevented STING translocation from the endoplasmic reticulum (ER) to the Golgi apparatus [71]. This finding positions GPX4 as an upstream regulator of STING trafficking. Excessive lipid peroxidation mediated by the STING signaling pathway contributes to intestinal ischemia-reperfusion injury. Inhibiting lipid peroxidation can prevent and treat STING pathway activation-induced intestinal ischemia-reperfusion injury and associated remote organ damage [72]. STING interacts with fatty acid desaturase 2 (FADS2), the key rate-limiting enzyme in the biosynthesis of n-6 PUFA arachidonic acid (AA) and n-3 PUFAs eicosapentaenoic acid (EPA) and docosahexaenoic acid (DHA) [73]. Within the ER, STING interacts with FADS2, inhibiting its enzymatic activity. Consequently, STING deficiency leads to increased levels of PUFAs, long-chain PUFAs (LC-PUFAs), and PUFA derivatives. Notably, STING and FADS2 exhibit significant structural similarity, suggesting that PUFAs and LC-PUFAs may function as STING ligands. Furthermore, PUFAs can reciprocally inhibit STING activity. This study elucidates the link between STING and lipid peroxidation from the perspective of PUFA synthesis regulation [73]. In acute myeloid leukemia (AML) cells harboring the RUNX1T1 (also known as AML1-ETO or AE) fusion gene, inhibiting STING releases FADS2 activity, thereby inducing PUFA synthesis and triggering lipid peroxidation-associated cell death [74]. In colon adenocarcinoma (COAD), the intracellular redox homeostasis maintained by GPX4 is crucial for activating the cGAS-STING signaling pathway. Conversely, terminal products of lipid peroxidation exert a specific inhibitory effect on the cGAS-STING pathway in COAD [75].

Mechanistic studies demonstrate that STING directly binds ACSL4 at residues D53 and K412, triggering ACSL4-driven ferroptosis, exacerbating nephritis and renal fibrosis. Critically, pharmacological or genetic inhibition of ACSL4 suppresses mtDNA-STING-mediated nephritis induced by angiotensin II (AngII), indicating that targeting the STING/ACSL4 axis could mitigate hypertension-related CKD pathogenesis [76].

### Cross-talk at the Intersection of GPX4 autophagic degradation

As summarized in Fig. 4, evidence revealed that STING promotes ferroptosis through GPX4 autophagic degradation. In myocardial ischemia-reperfusion injury (MIRI), STING drives ferroptosis by promoting autophagic degradation of GPX4. This process is mediated through a physical pairing of T267 (STING)-N146 (GPX4) residue, where STING facilitates autophagosome-lysosome fusion to initiate GPX4 degradation. The STING-GPX4 complex initially localizes to the ER-Golgi intermediate compartment (ERGIC) prior to autophagosomal translocation, and its formation is potentiated by cGAMP. This cGAMP-enhanced STING-directed autophagic degradation of GPX4 establishes a druggable target for MIRI therapy [77].

A study revealed STING upregulation in multiple sclerosis (MS) models, dissociating from endoplasmic reticulum calcium sensor STIM1 via glutamate excitotoxicity, leading to activation of non-canonical STING signaling, triggering GPX4 autophagic degradation and ferroptosis. Genetic and pharmacological targeting of neuronal STING protects against inflammation-driven neurodegeneration [78]. Conversely, in sepsis-induced acute kidney injury, inhibition of STING activation suppresses GPX4 autophagic degradation and ferroptosis [79]. In a study of pancreatic cancer, STING-driven GPX4 autophagic degradation induces autophagy-dependent ferroptosis [80].

### The cGAS-STING pathway triggers ferroptosis through intermediary mechanisms

**Fig. 5:**
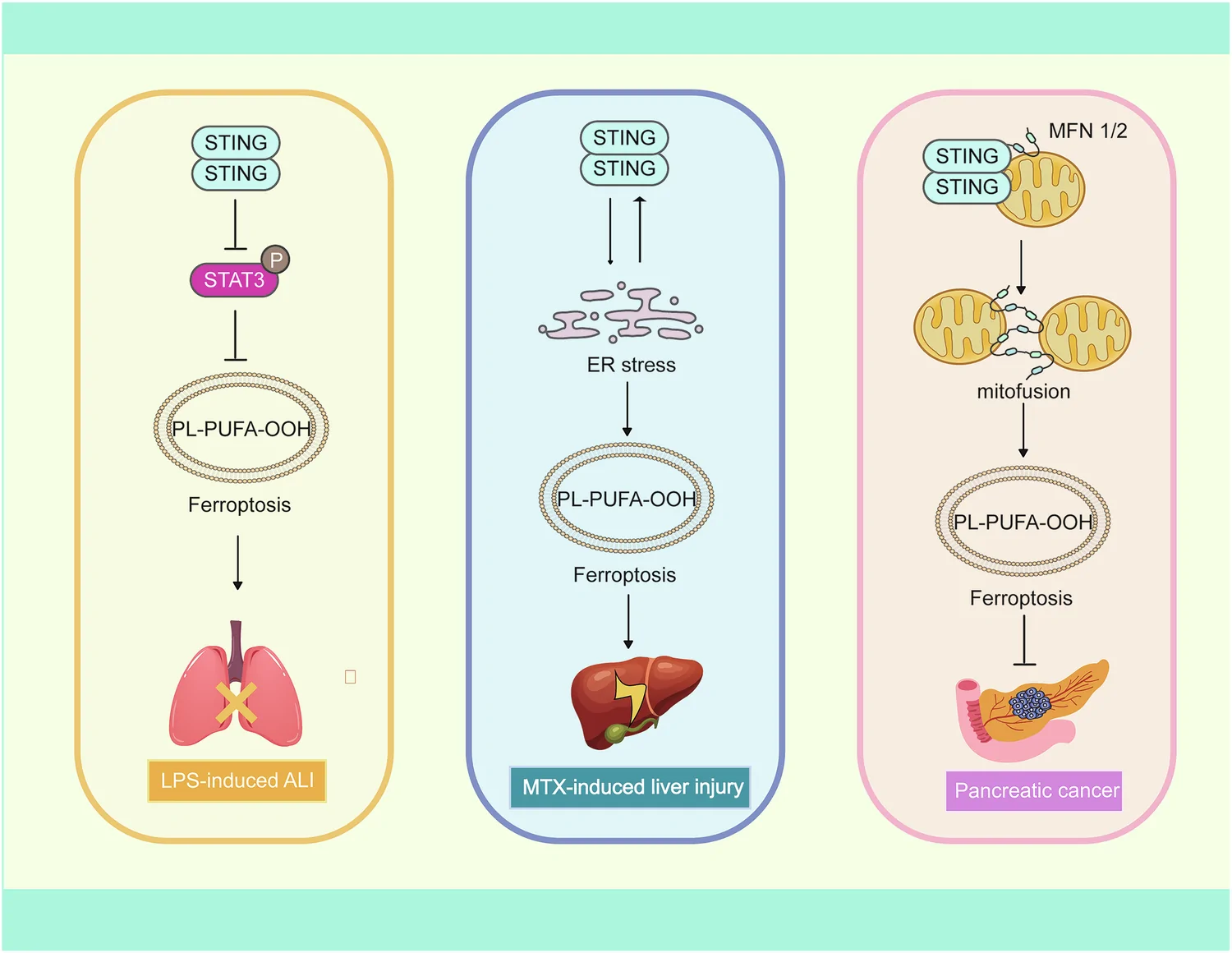
The cGAS-STING pathway contributes to ferroptosis indirectly. (1) In lipopolysaccharide (LPS)-induced acute lung injury, STING promotes ferroptosis and exacerbates lung damage by suppressing p-STAT3 levels. (2) The STING pathway aggravates methotrexate-induced liver injury by mediating endoplasmic reticulum stress-dependent ferroptosis. (3) In pancreatic cancer, STING binds to MFN1/2 and triggers MFN1/2-dependent mitochondrial fusion, thereby promoting ferroptosis in pancreatic cancer cells and suppressing tumor progression.

Research has unraveled that the cGAS-STING pathway is triggers through intermediary ways, such as endoplasmic reticulum (ER) stress, p-STAT3 and mitofusion, as shown in Fig. 5. The endoplasmic reticulum (ER) is crucial for ferroptosis. The ER lumen constitutes an oxidative environment where oxidative stress can induce ferroptosis [81]. ER stress can be triggered by ferroptosis inducers, such as System Xc⁻ inhibitors, consequently leading to ferroptosis [82]. This may partially stem from ER stress disrupting calcium (Ca²⁺) homeostasis, thereby inducing oxytosis—a cell death process considered mechanistically similar to ferroptosis [83]. Current research confirms that ER stress activates STING [84–86], an ER-resident protein. Conversely, STING also promotes ER stress [84], as observed during cellular responses to bacterial c-di-AMP [30, 87]. Recent studies further reveal that ER stress plays critical roles in STING-induced ferroptosis. Methotrexate (MTX), an antimetabolite widely used in anticancer chemotherapy, exhibits significant hepatotoxicity that limits its clinical utility. A study confirms that endoplasmic reticulum (ER) stress underlies MTX-induced liver injury and STING-mediated ferroptosis [88]. Targeting the STING–ER stress–ferroptosis axis may represent a promising therapeutic strategy against MTX hepatotoxicity.

In LPS-induced acute lung injury (ALI), STING knockout elevates p-STAT3 levels, attenuating inflammatory damage and ferroptosis. Conversely, STAT3 inhibition exacerbates ferroptosis post-STING ablation, indicating that STING regulates ferroptosis through the STAT3 signaling axis [89]. Collectively, STING mediates LPS-induced ALI by modulating ferroptosis, highlighting both STING and ferroptosis as novel therapeutic targets for ALI.

The ER-resident protein STING promotes ferroptosis in human pancreatic cancer cell lines by binding mitofusin 1/2 (MFN1/2) to trigger MFN1/2-dependent mitochondrial fusion rather than mitophagy. Genetic knockout of STING1 or MFN1/2 reduces ferroptosis sensitivity in vitro and in xenograft models, revealing a strategy to potentiate ferroptosis-based therapies [90].

## Implication in diseases

**Fig. 6:**
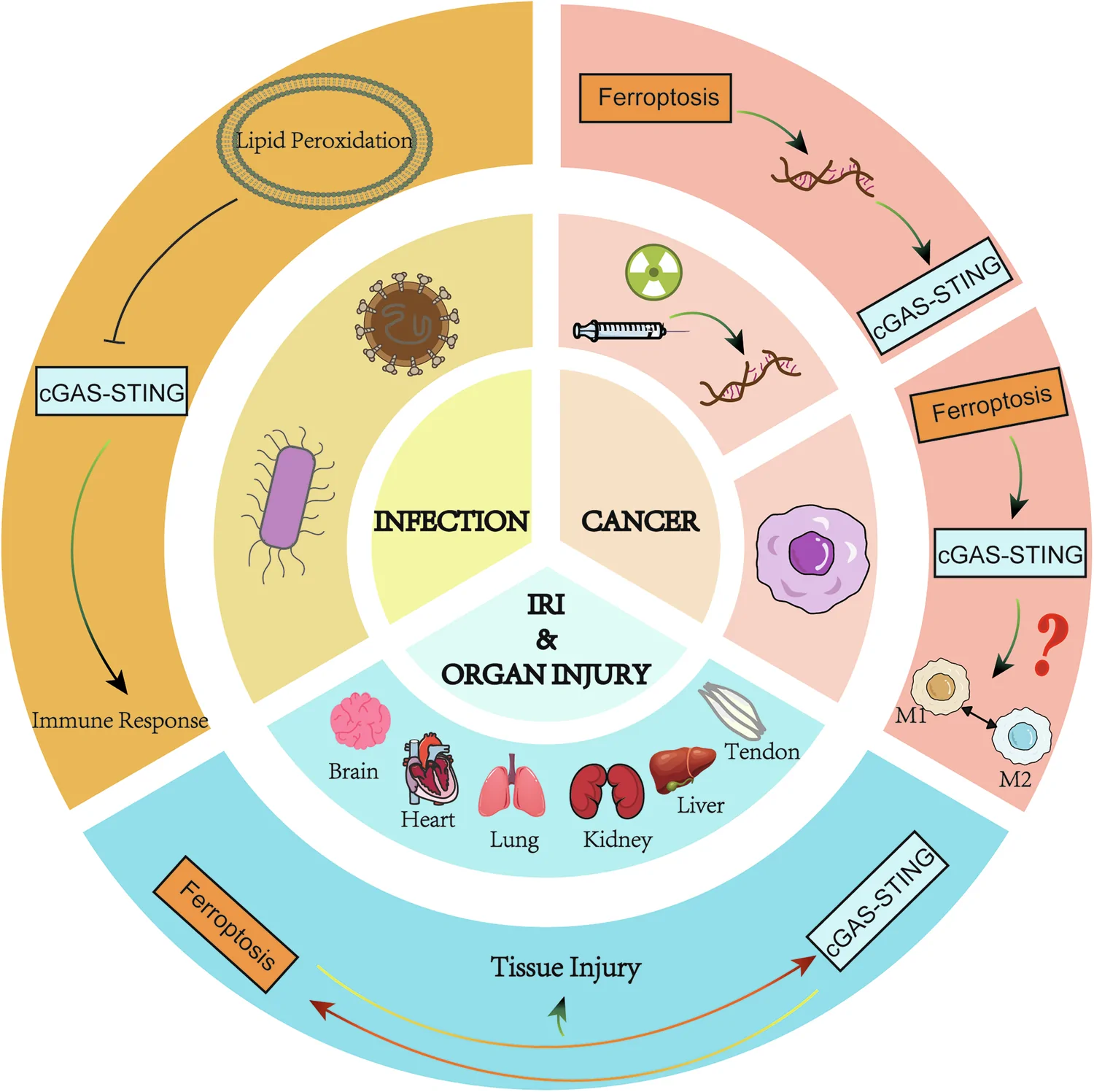
The interplay between the cGAS-STING pathway and ferroptosis in diseases. (1) In Cancer: Therapies such as radiotherapy and chemotherapy induce DNA damage, which activates the cGAS-STING pathway. Ferroptosis is also involved in this treatment-induced DNA damage process. Concurrently activating both ferroptosis and the cGAS-STING pathway can synergistically enhance tumor cell killing. Regarding the polarization of tumor-associated macrophages (TAMs) in the tumor microenvironment, the interaction between STING and ferroptosis is implicated; however, the direction of polarization they promote remains debated. (2) In Infectious and Inflammatory Diseases: In the context of infection, ferritinophagy and lipid peroxidation can suppress the activation of cGAS-STING. Inhibiting ferroptosis by targeting lipid peroxidation or iron metabolism not only alleviates tissue damage caused by ferroptosis but may also promote the cGAS-STING pathway, thereby enhancing immune defense against pathogens. (3) In Ischemia-Reperfusion Injury (IRI) and Organ Injury: A mutually reinforcing cycle exists between the cGAS-STING pathway and ferroptosis, exacerbating ischemia-reperfusion injury. Therapeutic strategies that inhibit lipid peroxidation and/or cGAS-STING activation can ameliorate organ damage following IRI.

As previously discussed, the established cGAS-STING pathway and ferroptosis offer novel therapeutic avenues for currently incurable diseases. We now discuss the confirmed and potential mechanistic interactions between STING and ferroptosis, alongside their implications for treating cancer, infections, and inflammatory diseases. And we concluded the role of interplay between STING and ferroptosis in diseases in Fig. 6.

### In cancer

STING expression is frequently lost in many tumor cells. This deficiency promotes premature activation of cyclin-dependent kinase 1 (CDK1) and increased chromosomal instability, driving cells into S-phase and mitosis prematurely. This mechanism may explain the association between STING loss and poorer prognosis in cancer patients [91]. Concurrently, cancer cells exhibit heightened iron dependence compared to normal cells and exploit PUFA-enriched microenvironments to enhance therapy resistance [2]. However, this strong iron reliance also renders them more vulnerable to ferroptosis and ROS accumulation, positioning ferroptosis-mediated cancer therapy and tumor microenvironment (TME) targeting as viable strategies [21].

As outlined, the interplay between the cGAS-STING pathway and ferroptosis has been demonstrated in various cancers. In PIN-promoted cervical cancer, ferroptosis is suppressed following inhibition of the cGAS-STING pathway [92]. In multiple tumor models ferroptosis can promote cGAS-STING pathway activation. This activation may foster tumor growth by mediating macrophage infiltration, activation, and migration [51, 56] or conversely, inhibit tumor growth via positive feedback loops that amplify ferroptosis [55]. Therapeutic agents exploiting this interplay have been developed [52–54, 57]. Simultaneous promotion of the cGAS-STING pathway and ferroptosis demonstrates synergistic anti-cancer effects [44]. Current primary cancer treatments include surgical resection, chemotherapy, radiotherapy, and immunotherapy. We elaborate on the role of STING-ferroptosis crosstalk in enhancing these modalities.

Radiotherapy and certain chemotherapeutic agents (e.g., cisplatin, etoposide) exert their effects partly through DNA damage, with both STING activation and ferroptosis contributing to their efficacy. Mechanistically, therapy-induced DNA damage generates micronuclei that accumulate in the cytoplasm, activating the cGAS-STING pathway [4]. Furthermore, in murine ovarian cancer cells, the cGAS-STING pathway activation is a mechanism underlying cisplatin-mediated cytotoxicity [93]. Intravenous administration of STING agonists (e.g., SB11285) synergizes with radiotherapy to enhance efficacy in HNSCC [91], highlighting the therapeutic potential of combining STING agonists with radiotherapy or DNA-damaging chemotherapy. Concurrently, ferroptosis contributes to the efficacy of radiotherapy and cisplatin. Radiotherapy induces ferroptosis via ACSL4 upregulation and SLC7A11 downregulation. Ferroptosis inducers (e.g., IKE, RSL3) synergize with radiotherapy by increasing lipid peroxidation and anti-tumor efficacy [94]. In cisplatin-resistant ovarian cancer cells, erastin potentiates cisplatin cytotoxicity [95]. Notably, impaired ferroptosis induction in tumor cells facilitates mitochondrial DNA (mtDNA) leakage into the cytosol, activating the cGAS-STING pathway. This mechanism offers significant potential for enhancing the efficacy of radiotherapy and DNA-damaging chemotherapy, including multifunctional exosomes disrupting intracellular iron metabolism and redox homeostasis (Exo@MnIO&BG) [52], a mtDNA-guided tumor immunotherapy nanoplatform (HBMn-FA) [53], and a novel radiosensitizer (Hafnium oxide-based) amplifying ferroptosis cascades to robustly activate the cGAS-STING pathway via damaged DNA and Mn²⁺ [54]. Exploiting targeted ferroptosis to further activate the cGAS-STING pathway holds promise for combination strategies with radiotherapy and DNA-damaging chemotherapy.

The TME critically influences tumor progression and therapy response. Tumor-associated macrophages (TAMs), key components of the TME, polarize into functionally distinct M1 (pro-inflammatory, anti-tumor) and M2 (anti-inflammatory, pro-tumor) phenotypes [96]. M2-TAMs promote tumorigenesis, metastasis, and immunosuppression by degrading the endothelial basement membrane, promoting angiogenesis, and inhibiting immune responses [97]. Reprogramming TAMs towards the M1 phenotype or reducing their infiltration are major strategies in cancer immunotherapy. In pancreatic cancer, the STING pathway promotes TAM polarization towards the anti-tumor M1 phenotype [97, 98]. Accordingly, DHA@MIL-101 was developed to activate the STING pathway via ferroptosis, promoting M1 macrophage polarization for tumor treatment [57]. Conversely, in PDAC tumor cells, ferroptosis promotes TAM polarization towards the pro-tumor M2 phenotype [51, 99]. Intriguingly, TMEM173 (STING) was identified as a mechanism involved in ferroptosis-driven M2 polarization [51]. Thus, while STING promotes anti-tumor M1 polarization in pancreatic cancer, it paradoxically participates in ferroptosis-induced M2 polarization. Further research is needed to clarify the roles of ferroptosis and the STING pathway in TAM polarization within the TME.

A positive feedback loop between cGAS-STING pathway effectors and ferroptosis [55], the promotion of ferroptosis by type I IFNs downstream of cGAS-STING, and the significant tumor growth inhibition achieved by combining IFN-β with RSL3 [44] collectively highlight the therapeutic potential of co-administering cGAS-STING agonists and ferroptosis inducers.

### In infectious and inflammatory diseases

#### Bacterial and viral infection

Viral and bacterial DNA activate cGAS, while CDNs directly activate STING, triggering the cGAS-STING pathway to mount immune defenses against pathogens. However, ferroptosis has been implicated in host tissue and cellular damage caused by bacteria like *Mycobacterium tuberculosis* and *Pseudomonas aeruginosa* [3, 100, 101]. Iron is crucial for viral replication; iron deficiency can enhance infection and cell death for some viruses, while viruses that increase intracellular iron or enhance intestinal iron absorption may induce ferroptosis [102]. Consequently, therapies targeting iron metabolism may mitigate exacerbated viral infections caused by ferroptosis [102]. Therefore, in certain bacterial and viral infections, simultaneously promoting the STING pathway and inhibiting ferroptosis might reduce host damage while strengthening pathogen clearance. Supporting this notion, as mentioned earlier in the context of infection, ferroptophagy [65] and lipid peroxidation inhibit cGAS-STING activation [65, 71]. This further validates the strategy that inhibiting ferroptosis (via suppressing lipid peroxidation or modulating iron metabolism) could not only alleviate ferroptosis-mediated damage but also enhance anti-pathogen immunity by promoting the cGAS-STING pathway.

#### Ischemia-reperfusion-injury(IRI) and organ injury

Ischemia-reperfusion injury (IRI) occurs when blood flow and oxygen supply are restored to an organ after a period of ischemia. Multiple cell death modalities, including autophagy [103], apoptosis [104], necroptosis [105] and ferroptosis [106], contribute to IRI. Following organ IRI, the release of damage-associated molecular patterns (DAMPs) [107], particularly mitochondrial DNA (mtDNA), activates the cGAS-STING pathway and its downstream signaling cascade, exacerbating inflammation [108]. Beyond its direct pro-inflammatory role, STING can also cooperate with IFN and TNF-α to induce necroptosis, further aggravating IRI [105]. Multiple studies demonstrate that the cGAS-STING pathway and ferroptosis mutually potentiate each other to worsen IRI, including in myocardial [50, 77], cerebral [61], and intestinal IRI [72]. Therefore, inhibiting both lipid peroxidation and cGAS-STING pathway activation represents a feasible approach to ameliorate organ IRI.

Evidence across various organ injuries suggests that the cGAS-STING pathway and ferroptosis often act synergistically to exacerbate damage:In sepsis, STING interacts with NCOA4 in activated macrophages, triggering ferritinophagy-mediated ferroptosis, which amplifies inflammation and organ injury [66]. Mutual potentiation between the cGAS-STING pathway and ferroptosis contributes to injury in TBI [41], neurodegenerative diseases [46, 47, 58, 78], and injuries of the heart [36, 39, 49], lung [35, 89], kidney [40, 64], liver [45, 88] and tendons [37, 38]. Collectively, this evidence strongly supports that dual inhibition of both the cGAS-STING pathway and ferroptosis holds significant promise for mitigating organ damage.

## Discussion

The intricate interplay between the cGAS-STING pathway and ferroptosis is rapidly reshaping research paradigms in cell death biology and disease pathogenesis. Integrated evidence presented in this review demonstrates their interaction via multiple mechanistic axes, including iron metabolism (NCOA4-mediated ferritinophagy, TFR1/FPN1-dependent iron transport), lipid peroxidation (the ACSL4/ALOXs-PUFA-PLOOH axis, FADS2-driven PUFA synthesis), and GPX4 autophagic degradation—ultimately culminating in lethal membrane damage and oxidative stress. Furthermore, STING can indirectly induce ferroptosis through ER stress, STAT3 signaling, and mitochondrial fusion. Notably, the discovery of mitochondrial outer membrane-localized cGAS reveals a non-canonical STING-independent ferroptosis suppression pathway mediated by DRP1 oligomerization, challenging the conventional view of cGAS solely as a cytosolic DNA sensor promoting ferroptosis.

The cGAS-STING-ferroptosis axis exhibits context-dependent properties in disease regulation. In cancer, synergistic activation of both pathways (e.g., STING agonists + ferroptosis inducers) exploits the vulnerability of cancer cells to iron overload and lipid peroxidation, enhancing the efficacy of immunotherapy/radiotherapy. Nanoplatforms (e.g., HBMn-FA52, Exo@MnIO&BG51), by co-targeting mtDNA-cGAS-STING activation and iron-redox homeostasis, demonstrate significant clinical translational potential. In infectious diseases, the occurrence of ferroptosis suppresses STING-mediated immune defence. Conversely, inhibiting ferroptosis while promoting the cGAS-STING pathway strengthens host defence against viral, bacterial, and other infections. As for organ injury and IRI, mutual potentiation of cGAS-STING and ferroptosis exacerbates tissue damage. Inhibiting ferroptosis while preserving STING-mediated immune defence emerges as a novel strategy to balance host protection and tissue injury. However, unresolved complexities remained in pancreatic cancer. STING exhibits dual roles, not only promoting anti-tumor M1-polarization of TAMs but also paradoxically participating in ferroptosis-mediated M2-polarization of TAMs. This highlights significant unexplored mechanistic territory.

Emerging molecular hubs interconnect cGAS-STING and ferroptosis. Although associations between the pathways have been increasingly recognized over the past 5 years, numerous potential links remain unexplored. NRF2 is implicated as a potential coordinator. NRF2 inhibits ferroptosis by transcriptionally activating antioxidant genes (SLC7A11, FTH1/FTL, GPX4) [109] and negatively regulates STING expression by reducing STING mRNA stability [110]. This dual negative regulation (e.g., swim training suppressing both pathways via miR-17-3p/KEAP1/NRF2 [49]) suggests NRF2 may be a key node balancing their interaction, though its direct regulatory mechanisms on cGAS-STING require elucidation. Beclin-1, encoded by BECN1, a key autophagy regulator, promotes ferroptosis by forming a Beclin-1-SLC7A11 complex [111] and facilitating ferritinophagy [112–114], yet suppresses the STING pathway by interacting with cGAS to inhibit cGAMP synthesis [22, 111]. HSP90, primarily existing as homodimers, promotes ferroptosis by positively regulating chaperone-mediated autophagy (CMA) via stabilizing the CMA receptor LAMP2A, leading to GPX4 degradation [115]. Conversely, its isoform HSP90β enhances STING protein stability [116], marking it as a potential dual-pathway target. STAT3 exhibits context-dependent regulation. In PDAC, p-STAT3(Tyr705) activates STING via the lysosome-nuclear DNA damage axis and induces GPX4 degradation-dependent ferroptosis [80]. However, STAT3 itself suppresses ferroptosis by binding to the SLC7A11/GPX4 promoters [117–119] and inhibits STING immune responses via IL-6 feedback [120].

The STING-ferroptosis interface is a dynamic frontier. Mechanistic discoveries within this field continuously reshape therapeutic landscapes. Harnessing its potential requires precise navigation of its dual nature—augmenting cytoprotection or cytotoxicity based on disease context. Future breakthroughs will hinge on deciphering how microenvironmental signals regulate this balance, ultimately paving the way for spatiotemporally precise interventions.
